# The development and evaluation of a computerized diagnosis scheme for pneumoconiosis on digital chest radiographs

**DOI:** 10.1186/1475-925X-13-141

**Published:** 2014-10-02

**Authors:** Biyun Zhu, Wei Luo, Baoping Li, Budong Chen, Qiuying Yang, Yan Xu, Xiaohua Wu, Hui Chen, Kuan Zhang

**Affiliations:** School of Biomedical Engineering, Capital Medical University, Beijing, 100069 China; Department of Radiology, Coal General Hospital, Beijing, 100028 China; Department of Radiology, Beijing Friendship Hospital, Capital Medical University, Beijing, 100053 China; Beijing Key Laboratory of Fundamental Research on Biomechanics in Clinical Application, Capital Medical University, Beijing, 100069 China

**Keywords:** Digital radiograph, Pneumoconiosis, Bootstrap resampling, Texture feature, Support vector machine

## Abstract

**Purpose:**

To diagnose pneumoconiosis using a computer-aided diagnosis system based on digital chest radiographs.

**Methods:**

Lung fields were first extracted by combining the traditional Otsu-threshold method with a morphological reconstruction on digital radiographs (DRs), and then subdivided into six non-overlapping regions (region (a-f)). Twenty-two wavelet-based energy texture features were calculated exclusively from each region and selected using a decision tree algorithm. A support vector machine (SVM) with a linear kernel was trained using samples with texture features to classify an individual region of a healthy subject or a pneumoconiosis patient. The final classification results were obtained by integrating these individual classifiers with the weighted voting method. All models were developed on a dataset of 85 healthy controls and 40 stage I or II pneumoconiosis patients and validated by using the bootstrap resampling with replacement method.

**Results:**

The areas under receiver operating characteristic curves (AUCs) of regions (c) and (f) were 0.688 and 0.563, which were worse than those of the other four regions. Region (c) and (f) were both excluded from the individual classifiers that were going to be assembled further. When built on the selected texture features, each individual SVM showed a higher diagnostic performance for the training set and the test set. The classification performance after an ensemble was 0.997 and 0.961 of the AUC value for the training and test sets, respectively. The final results were 0.974 ± 0.018 for AUC value and 0.929 ± 0.018 for accuracy.

**Conclusion:**

The integrated SVM model built on the selected feature set showed the highest diagnostic performance among all individual SVM models. The model has good potential in diagnosing pneumoconiosis based on digital chest radiographs.

## Introduction

Pneumoconiosis is a serious occupational disease worldwide with high incidence caused by inhaled particles such as free silica, asbestos, mixed dust, coal, beryllium, and cobalt [1]. The International Labor Organization (ILO) has established a standardized system for classifying radiographic abnormalities of pneumoconiosis [2]. Digital chest radiography is the most practical tool for lung disease diagnosis. On chest radiographs, silicosis appears as small rounded and irregular opacities while asbestosis appears as small irregular opacities. Compared with ILO standard radiographs, a radiologist assesses the concentration of small opacities of a chest X-ray image as category 0, I, II, or III [2]. The higher the category, the more serious the condition. Because categories I and II typically indicate small nodular and irregular opacities on chest radiographs, it is difficult for radiologists to classify them as pneumoconiosis [3]. Therefore, many investigators devoted themselves to developing computer-aided diagnosis (CAD) schemes based on chest radiographs, which were necessary to reduce workloads and improve workflow in mass chest screening.

In medical images, changes of texture features often reflect the pathological changes of the body. Therefore, texture analysis of medical images is of great significance for the differential diagnosis of diseases. Yu *et al*. [4–6] detected pneumoconiosis using feature vectors gained through a gray-level histogram co-occurrence matrix on digital radiographs (DRs). Katsuragawa *et al*. [7] developed a texture analysis method based on a geometric pattern feature analysis method to detect interstitial infiltrates in digital chest radiographs. A texture analysis method of liver CT images based on a spatial gray-level dependence matrix, a gray-level run length method, and a gray-level difference method has been proposed [8].

From a machine learning point of view, it is a key to determine if a subject has pneumoconiosis, which is a binary classification problem. The support vector machine (SVM) is a popular machine learning model for binary classification, and is used in many fields as a powerful classification tool. Zhu *et al*. [9] differentiated benign and malignant pulmonary nodules based on an SVM with a Gaussian kernel to evaluate the performance of a classifier by comparing the results of an SVM-based classifier and a model based on artificial neural networks (ANN). The results showed that the SVM-based classifier had a better classification performance. Yuan *et al*. [10] used SVM to implement cancer diagnosis and evaluate the prognosis of breast cancer patients, with an accuracy of 96.24%, which was superior to those of other classifiers including K-Nearest Neighbor, Probabilistic Neural Network, and Decision Tree (DT). Lu *et al*. [11] proposed a topic identification approach to identify topics automatically of the health-related messages. In terms of classification performance, an SVM model outperformed DT and Naïve Bayes classifiers.

In this study, we extracted energy texture features from digital chest radiographs, which were used after feature selection as the input of an SVM-based classifier. Final SVM classifier was the ensemble of individual classifiers built dependently on individual lung regions.

### The CAD system

**Figure 1:**
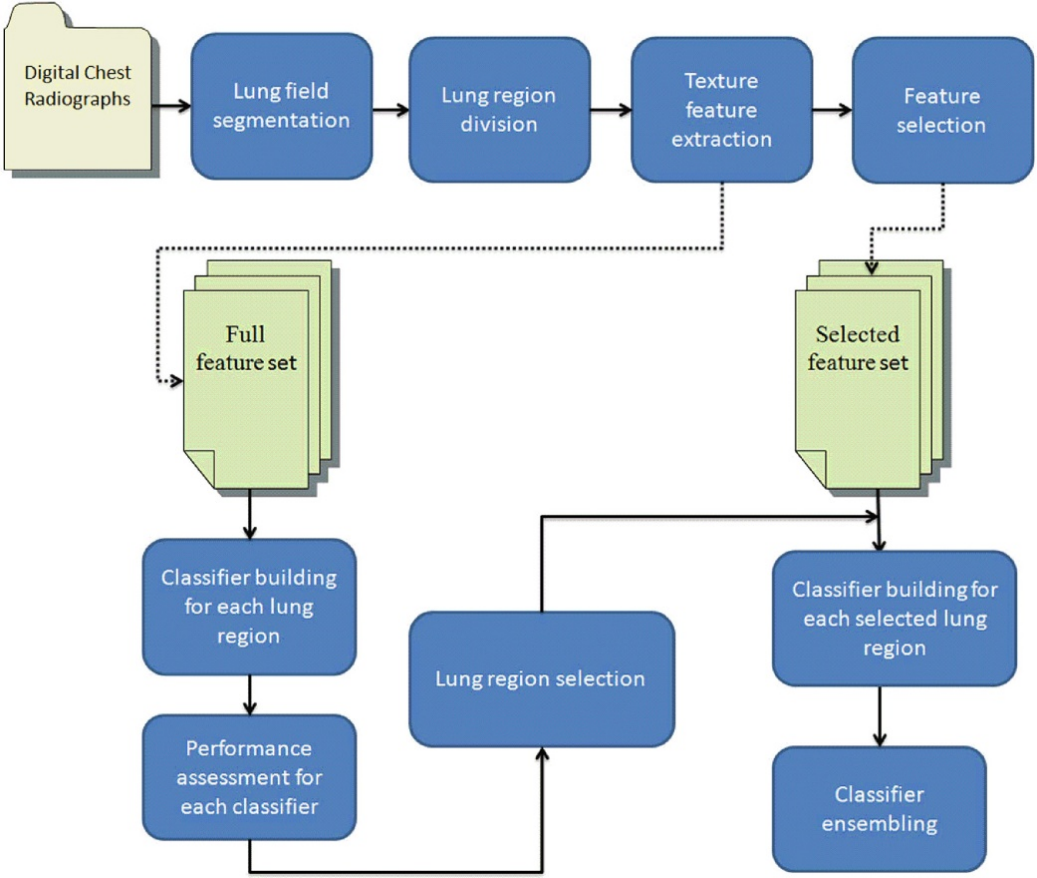
**Research procedure of the computerized diagnosis scheme described in our study.**

The CAD system for pneumoconiosis is based on the SVM algorithm. There are four main components involved to develop the CAD scheme: lung segmentation and subdivision, feature extraction and selection, classifier establishment and ensemble, and model performance validation. The flowchart of the procedure is shown in Figure 1.

The segmentation of lung fields was a preprocessing step in developing the CAD system for chest images to reduce the background interference outside lung tissues. The segmentation was performed by using the combination of the Otsu-threshold method with morphological reconstruction. Lung fields were then divided into six regions of interest (ROIs). Secondly, a discrete wavelet transform was employed prior to extracting the energy texture features for images in each subdivided region. The calculated features were then selected by a decision tree algorithm. Thirdly, six individual SVM models with a linear kernel were trained respectively using the feature set derived from each lung region to classify the ROI of a healthy subject or of a pneumoconiosis patient. SVM models built on six lung regions were assembled to obtain a classification result for the whole image by weighted voting. The classification models were evaluated by using a 0.632 bootstrap method.

### Lung segmentation and subdivision

**Figure 2:**
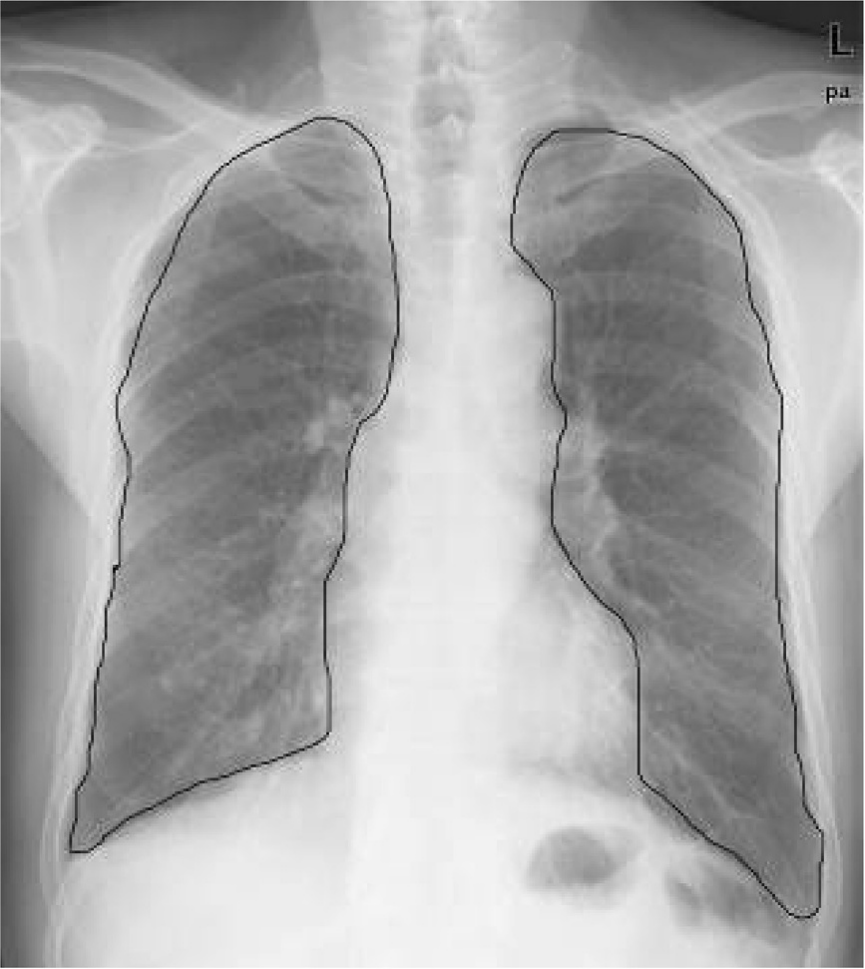
**Results of lung field segmentation using the Otsu**-**threshold algorithm based on morphological reconstruction.**

Different from other medical images such as CT scans or MR images, a chest radiograph may contain a lot of noise with various degrees of overlap among different organs, which can have an adverse effect on lung field extraction. Therefore, we presented a proven algorithm based on the Otsu-threshold method and extracted intact lung fields using a connected components labeling technique in our previous study [12]. An example result of lung field segmentation is shown in Figure 2. If the proposed method didn’t provide perfect segmentations for some digital radiographs (up to about 10% of all DR images), the corrected lung contours were drawn manually by radiologists.

Radiologists’ clinical practice showed that the first and most intuitive radiological symptom of digital chest radiographs for pneumoconiosis is the appearance of small opacities. The diagnosis guidance for pneumoconiosis developed by the ILO divides each lung field into three zones: the high lung field, middle lung field and low lung field, and the number density and area density of the small opacities are calculated in each of the six lung regions. Categories 0 ~ III have then been defined according to the profusion of small opacities [2, 13]. To make our study more clinically meaningful the lung fields were also divided into six zones. Feature extraction was applied in each region and each region was analyzed with a separate classifier based on these extracted features.

**Figure 3:**
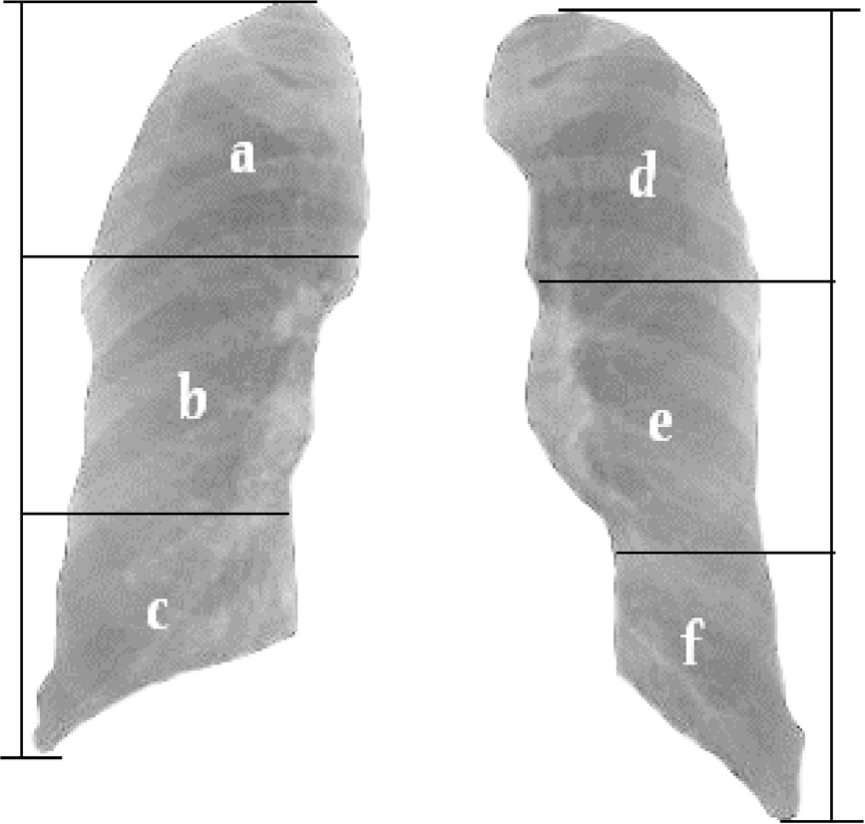
**The subdivision of lung fields.** The left and right lung are divided into six lung regions marked **(a)** to **(f)**. Lung region **(a-c)** correspond to upper, middle and lower lung field of the right lung, and region **(d-f)** correspond to upper, middle and lower lung field of the left lung, respectively.

The procedure of lung field division was as following: the vertical distance between the apex and diaphragm was calculated, then the positions of horizontal lines that divided each lung field into three regions with equal height were calculated, as shown in Figure 3.

### Feature extraction and selection

#### Feature extraction

The method of texture analysis is a two-dimensional discrete wavelet transform (DWT). Wavelet transform can capture both frequency and spatial information and has merits of multi-resolution and multi-scale decomposition [14, 15]. After decomposition for the first time, the original image is divided into four sub-band images of equal size of that quarter. These four images are *LL*_*i*_, *LH*_*i*_, *HL*_*i*_ and *HH*_*i*_, which represent the *i*^th^ scale. The sub-band *LL*_*i*_ represents the low-frequency sub-image, corresponding to an approximation image, which is to be decomposed in the (*i* + 1)^th^ scale; the sub-bands *LH*_*i*_, *HL*_*i*_ and *HH*_*i*_ collectively called low-frequency sub-images correspond to detail images. In first-level decomposition, the size of the original image of size *M* × *N* is decomposed into four sub-bands *LL*_1_, *LH*_1_, *HL*_1_ and *HH*_1_, of size *M*/2 × *N*/2. After the *i*^th^-level decomposition, the size of the three sub-bands *LH*_*i*_, *HL*_*i*_ and *HH*_*i*_ is *M*/2^*i*^ × *N*/2^*i*^[16].

**Figure 4:**
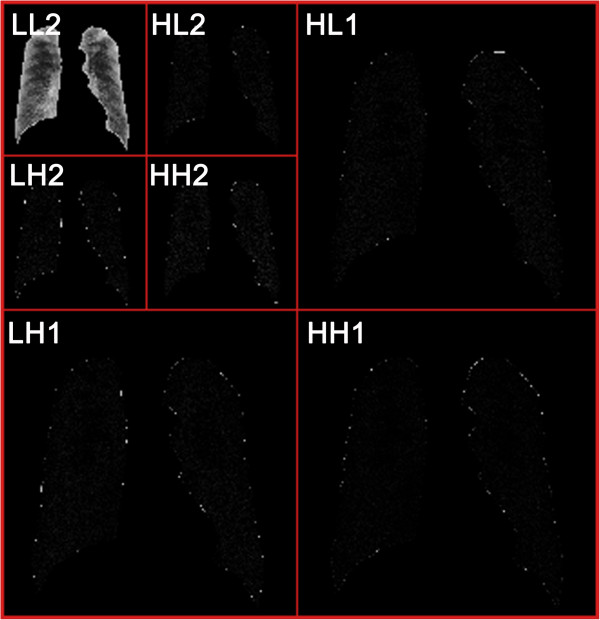
**Illustration of the first two wavelet decompositions of a seven**-**scale wavelet transform of a lung field image**, **resulting in 7 sub**-**bands.**

The selection of the wavelet base is very important in practical applications of DWT. When analyzing the same problem, a different wavelet basis will produce a different result. So far, there has been no good way or a unified standard to solve the problem, and the main method used is to determine a wavelet base that is applicable for a problem according to the error between the results derived from wavelet analysis and the theoretical results. In our study, we selected the well-known Daubechies as the wavelet base, which may provide a more effective analysis than others in the scenario of image processing. Daubechies 7 (db7) was determined by trial-and-error. Taking the resolution of the given images used in our study into consideration, each DR image has been wavelet-decomposed seven times (a seven-scale wavelet transform), resulting in 22 sub-bands of each image (i.e., *LH*_1_, *HL*_1_, *HH*_1_ … *LH*_6_, *HL*_6_, *HH*_6_, *LL*_7_, *LH*_7_, *HL*_7_, *HH*_7_). Figure 4 shows the first two wavelet decompositions of the seven-scale wavelet transform of a lung field image.

The commonly adopted texture measure indicators based on 2-D DWT are L_1_ norm, energy, and entropy [17]. Results obtained from our previous study [18] showed that the energy indicator had a higher performance for diagnosing pneumoconiosis, and was thus adopted in the present study. In the seven-scale wavelet transform for each DR image, the energy *E*_*i*,l_ for the *l*^th^ sub-band image at the *i*^th^ scale (*l* = 1 to 4 and *i* = 1 to 7) is calculated as follows:
1

where *M* and *N* represent the size of the original DR image and *x*(*s*, *t*) is the gray value of pixel (*s*, *t*) of the image.

#### Feature selection

Feature selection is one of the key issues in pattern recognition, and the accuracy and generalization capability of a classifier are affected directly by the result of feature selection. Hence, it is crucial to study the effective methods of feature selection. Presently, there are many ways to conduct feature selection, such as Principle Component Analysis and the Relief and Genetic Algorithm [19, 20]. In this paper, feature selection was accomplished by the DT algorithm, which is well known as a kind of classification method rather than a feature selection method [21, 22].

**Figure 5:**
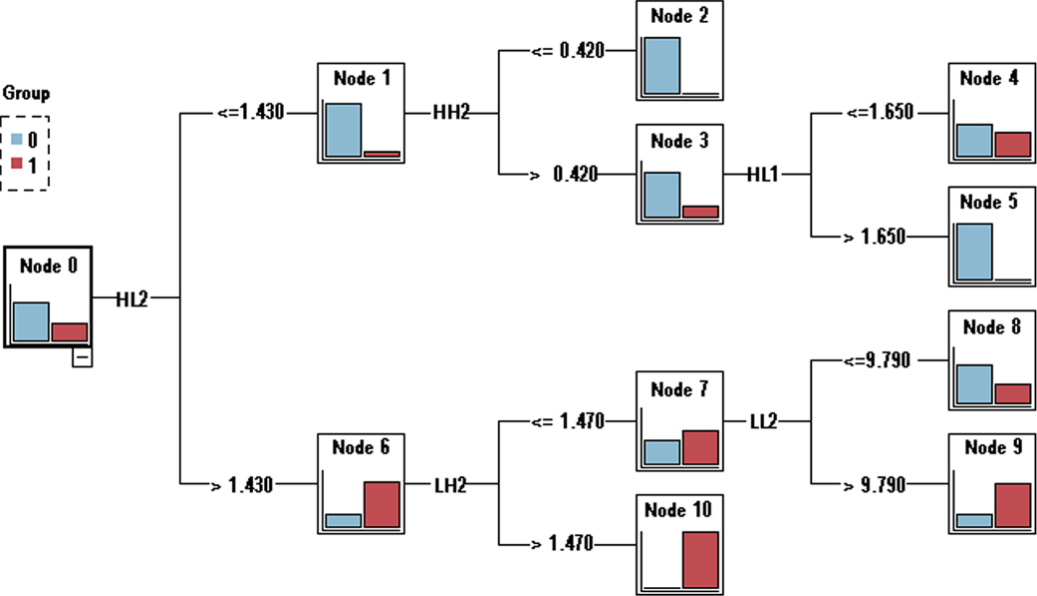
**An example of a decision tree for the feature selection.** Five energy features (log-transformed) are selected from a full set of seven features. There are six branch rules (corresponding to six leaf nodes) derived from the DT involving features extracted from five sub-bands, i.e. sub-band HL_2_, HH_2_, HL_1_, LH_2_ and LL_2_.

Essentially, the DT algorithm is a process of splitting a dataset into several subsets with the best classification ability based on those features that may satisfy some conditions. Then, every subset is refactored by these features. This process is repeated until all subsets contain only elements of a homogeneous type or the sample size included in each subset is smaller than some threshold. In addition to the classification results, another outcome of the DT algorithm is the features involved in the dataset splitting. These features can then be regarded as the selected features with the best classification performance in each subset. Therefore, the DT algorithm can be used to pare down the features, and the classifier will be built on the remaining features [23]. Figure 5 gives an example of a decision tree for selecting 5 energy features (log-transformed) from a full set of 7 features obtained after the first two wavelet decompositions of a lung field image shown in Figure 4.

DT with algorithm C5.0 splits the dataset according to features that can obtain the greatest information gain, and the splitting will cease when the number of samples to be split is below a certain threshold. It makes the DT suitable for and robust to handle numeric features and some missing values. In consideration of this, DT with algorithm C5.0 was chosen as the method of feature selection in our study.

### Classification based on lung regions

In the third stage, six individual SVMs with a linear kernel performed the classification tasks using the feature sets selected from each subdivided region in each image.

The SVM was first introduced by Vapnik in the mid-1990s based on the structural risk minimization principle, whose aim was to construct a hyperplane that maximizes the margin between negative and positive examples. The SVM performs well in small sample sizes and in high dimensional spaces [24].

Given the training set (*x*_*i*_, *y*_*i*_), where *x*_*i*_ ∈ *R*^*m*^ is an m-dimensional input vector (*i* = 1, …, *m*), and *y*_*i*_∈{−1, 1} is the label of *x*_*i*_. SVM finds the decision surface by solving the following optimization problem:
2

subject to:
3

where *w* is a weight vector and *b* is bias. The variables *ξ*_*i*_ are positive slack variables, which is necessary to allow misclassification. The parameter *C* is a penalty value, which seeks to penalize the decision errors when searching for the maximum marginal hyperplane. For a training sample *x*, the decision function can be given by
4

where *Φ* is the mapping function, which can make the linear learning machine work well in a non-linear case by mapping the original input space into a high dimensional feature space. The mapping function is defined by the kernel function: *K*(*x*_*i*_, *x*_*j*_) = *φ*(*x*_*i*_)^*T*^*φ*(*x*_*j*_). The simplest kernel in SVM is the linear kernel, which is defined as:
5

For an SVM with a linear kernel, the penalty factor *C* was the only fixed regularization parameter. It was determined experimentally as 1000 in our work.

### Classifiers ensemble

The final diagnosis result for the CAD scheme is based on the prediction result of the SVM model per region. In addition, the combination of the six classifiers can achieve a higher classification performance. However, one of the key problems of a classifiers ensemble is how to set the weight of each model. In this study, the weighted voting method was used to incorporate the regional scores into a stand-alone result.

The weight *w*_*i*_ for region *i* (*i* = 1, …, *m*) is determined by *A*_*vi*_ (the AUC value of region *i*) obtained from the training phase:
6

The final probability of a DR image being abnormal *P*_*ab*_ is given by
7

where *P*_*i*_, ranging from 0 to 1, is the output of the SVM-based classifier for lung region *i*, corresponding to the probability of a DR image being abnormal according to the texture feature of region *i*.

## Performance assessment and model validation

### Performance assessment

In this study, classification accuracy (Acc), sensitivity (Sen), specificity (Spe), and AUC were used as indicators to test the performance of the proposed SVM model. Acc, Sen and Spe are defined as follows:
8910

where *TP*, *TN*, *FP* and *FN* denote the number of abnormal cases that were correctly classified as pneumoconiosis, the number of normal cases correctly classified as healthy persons, the number of normal cases incorrectly classified as pneumoconiosis, and the number of abnormal cases incorrectly classified as normal persons.

AUC is the area under the receiver operating characteristic curve, which is constructed by plotting pairs of Sen and one minus Spe. AUC can measure the discrimination ability of the prognostic assessments. For our study, the larger the AUC, the better the ability of the CAD to discriminate DRs with pneumoconiosis from the normal DRs [25].

### Model validation

The classification performance of each SVM model was validated by a 0.632 bootstrap in our work.

The bootstrap method was first proposed by Efron in 1979 and usually used when the sample size is small. The process of the bootstrap method is to sample a dataset of *n* instances *n* times with replacement. For a particular instance, it has a probability of 1 − (1/n) of not being picked in a resampling, and therefore, has the probability of not being picked in total *n* times resampling as:
11

Then the approximately 36.8% of the samples which have never been picked will be used as the testing data to validate a classifier, and 63.2% of samples which have been picked at least once as the training data. The test data and training data do not overlap each other. This means that the testing data will contain approximately 36.8% of the instances and 63.2% for the training data [26, 27].

For *k*^th^ resampling with replacement, the indicator of performance evaluation, Sen, Spe, Acc and AUC, is given by:
12

Where *R*_test_^(*k*)^ was one of the above performance indicators for the testing data, *R*_train_^(*k*)^ for the training data, and *R*_final_^(*k*)^ for the total data for the *k*^th^ resampling, respectively. In this study, *k* was set to 100.

After resampling with replacement 100 times, the final classification result was averaged by repeating the process 100 times with different replacement samples for each classifier.

### Statistical methods

The databases were assigned to two groups: normal (*n* = 85) and the DRs with pneumoconiosis (*n* = 40). The texture features were extracted by using Matlab R2008a (The Mathworks Inc., Natick, MA, USA). The SVM models were implemented by using IBM SPSS Modeler 14 (IBM Corp., Armonk, NY, USA). The ROC analysis was performed by using ROCKIT (Charles E. Metz, Department of Radiology, University of Chicago, USA).

## Experiment and results

### Data description

We performed our procedure on 125 DRs collected from the Beijing Friendship Hospital, which were composed of 85 posterior-anterior digital chest radiographs for male normal subjects of average age of 62 years old and 40 for male pneumoconiosis patients of average age of 61 years old, of which 20 were stage I and 20 were stage II. The diagnostic decision of all the 40 pneumoconiosis patients was made by an radiologist panel. All images were digitized with a matrix of 2900 × 3000 and 15-bit gray level in the Digital Imaging Communications in Medicine (DICOM) format. The work was approved by the Beijing Friendship Hospital Research Ethics Committee.

### Classification performance of six individual models

#### Performance of SVM classifiers built on the full feature set

For each lung region, 22 energy features of wavelet coefficients were calculated after seven times wavelet decomposing, forming the full feature set. Upon the full feature set, one SVM with a linear kernel was built for each region. After repeating resampling with replacement 100 times, the classification performances were obtained by averaging the results of the 100 bootstrapped samples (see Table 1). It was obvious that the SVM built on features derived from region (c) had an almost perfect classification performance on the training dataset and a relatively poor classification performance on the test dataset, indicating the existence of an overfit. For the SVM from region (f), it had too low a classification performance on the test dataset, suggesting that this SVM was lacking the ability of generalization. Therefore, SVM classifiers built from region (c) and (f) were both excluded from the individual classifiers which were going to be assembled further.

**Table 1:** **Classification performance of the individual classifier for each lung region using the full feature set**

Lung region	Training dataset				Test dataset			
	AUC	Spe	Sen	Acc	AUC	Spe	Sen	Acc
(a)	0.932	0.885	0.852	0.877	0.855	0.805	0.776	0.791
(b)	0.961	0.991	0.682	0.891	0.884	0.928	0.699	0.826
(c)	0.996	1.000	0.976	0.990	0.688	0.788	0.474	0.682
(d)	0.824	0.833	0.655	0.778	0.742	0.763	0.594	0.710
(e)	0.924	0.902	0.766	0.854	0.834	0.838	0.699	0.791
(f)	0.722	0.570	0.791	0.648	0.563	0.531	0.728	0.592
Mean	0.893	0.864	0.787	0.840	0.761	0.776	0.662	0.732
SD	0.093	0.144	0.107	0.106	0.111	0.121	0.100	0.080

*AUC* = area under ROC curve; *Spe* = specificity; *Sen* = sensitivity; *Acc* = accuracy; *SD* = standard deviation.

Lung regions (a-c) correspond to upper, middle and lower lung field of the right lung, and region (d-f) correspond to upper, middle and lower lung field of the left lung, respectively.

#### Performance of SVM classifiers built on the selected feature set

The DT algorithm was performed to form the selected feature set from the full feature set, using training data of regions (a), (b), (d), and (e). The classification performance of the SVM which was built on the selected feature set is shown in Table 2.

**Table 2:** **Classification performance of the individual classifier for region** (**a**), (**b**), (**d**), **and** (**e**) **and the integrated using the selected feature set**

Lung region	Training dataset				Test dataset			
	AUC	Spe	Sen	Acc	AUC	Spe	Sen	Acc
(a)	0.986	0.832	0.894	0.852	0.879	0.753	0.844	0.786
(b)	0.997	0.996	0.737	0.910	0.939	0.934	0.738	0.866
(d)	0.953	0.811	0.724	0.782	0.835	0.762	0.623	0.711
(e)	0.994	0.971	0.800	0.913	0.889	0.880	0.697	0.817
Integrated	0.997	0.995	0.913	0.969	0.961	0.933	0.849	0.905

*AUC* = area under *ROC* curve; *Spe* = specificity; *Sen* = sensitivity; *Acc* = accuracy.

Lung regions (a) and (b) correspond to upper and middle lung field of the right lung, and region (d) and (e) correspond to upper and middle lung field of the left lung, respectively.

As the results indicated, the classification performances were improved in terms of accuracy and the AUC value for all SVMs from regions (a), (b), (d), and (e) on the selected feature set. For the training dataset, all AUC values were greater than 0.95, where some of them approached one, the perfect classification performance. In addition, the best sensitivity among the four regions for the training dataset was 0.894. The AUC value and Sen provided essential information for further validation on the test dataset. For the test dataset, the best AUC value of 0.939, Spe of 0.934 and Acc of 0.866 were found in region (b), whereas the maximum Sen of 0.844 in region (a). All these numbers showed a great potential in the integration of the four individual classifiers.

### Classification performance of the ensemble classifier

The weighting factors (0.251 for region (a), 0.254 for region (b), 0.242 for region (d), and 0.253 for region (e)) were determined by the AUC values (as listed in the first left column in Table 2) of the training dataset on the selected feature set according to Eq. (6). Diagnostic performance of the integrated SVM classifier, whose output was the weighted sum of each individual SVM classifier’s output, built on training dataset and test dataset are listed in the last row of Table 2.

The overall performance in terms of AUC, Sen, Spe and Acc of the integrated classifier were 0.974 ± 0.018, 0.957 ± 0.021, 0.873 ± 0.024 and 0.929 ± 0.018, respectively. No classification performance of an individual SVM built on an individual lung region was superior (P = 0.037, 0.603, 0.0004 and 0.087, respectively) with respect to the performance measure of AUC (0.918 for SVM built on region (a), 0.960 for region (b), 0.878 for region (d) and 0.928 for region (e)).

## Discussion

The CAD for pneumoconiosis has attracted many researchers in the past three decades. Yu *et al*. [4]. reported an accuracy of 87.7–89.2% and an AUC of 0.948–0.978. Xu *et al*. [28]. distinguished 175 digital pneumoconiosis images from 252 normal ones by evaluating gray-level co-occurrence matrix based features, with an accuracy of 95.5%. In addition to the texture characteristics of the image, Okumura *et al*. [29]. used the power spectrum of lung DRs after Fourier transformation, and the AUC for the detection of pneumoconiosis was 0.972. In our previous study [30], the use of texture features extracted through a gray-level histogram and co-occurrence matrix of chest DRs and artificial neural network with the back propagation algorithm also suggested a relatively high performance for the diagnosis of pneumoconiosis. Moreover, a few investigators detected pneumoconiosis by recognizing the small opacities on DRs according to the shape and size of the ROIs. Kondo *et al*. [31] trained an ANN to recognize small opacities on DRs including 12 pneumoconiosis images and 11 normal ones, showing that the method could identify the majority. The different results reported in these studies showed that the classification performance depends on every link of the CAD scheme, such as the database used, the features extracted and selected, the classifier chosen, and even the validation method when evaluating system performance.

As to the dataset used, almost all of these developed computerized schemes had one thing in common, i.e., the DR samples used in those studies included pneumoconiosis DRs of stage III patients. However, it is not difficult to make a diagnostic decision on stage III pneumoconiosis based on DRs for the clinician. To build a more clinically meaningful CAD model for pneumoconiosis, DR samples of pneumoconiosis patients of stage III were excluded from our study. In this scenario, the diagnostic performance for the developed CAD scheme in our study still reached a relatively high level with an accuracy of 92.9% and an AUC value of up to 0.974. It was neither inferior nor superior to those developed using DR samples including pneumoconiosis DRs of stage III in the literature referenced previously. Therefore, the CAD scheme developed in this study had potential for early diagnosis of pneumoconiosis.

With respect to the classifier chosen, the SVM is a popular learning model for application in binary classification, and performs better when dealing with multiple dimensions and continuous features. The classification performance of SVM is closely related to the choice of kernel function. In this study, SVM with a linear kernel for each region based on the selected feature set performed better than ones with a radial basis function kernel, sigmoid kernel, or polynomial kernel. In one of our previous studies [18], using an SVM with a polynomial kernel on full lung fields had the best performance, with an accuracy of 92.0% and an AUC value of 0.970. In this study, we used an integration method to ensemble individual classifiers into a single classifier. According to the ILO guidance and a radiologist’s diagnostic measurement, the lung field on DRs may be divided into six regions to reduce the influence of superimposed anatomical structures, which tend to make the abnormalities difficult to distinguish. Some studies have proven that the small opacities, especially small rounded opacities, on DRs of stage I and II pneumoconiosis patients are mainly distributed in the high and middle lung fields [32–34], which correspond to regions (a) and (d) and (b) and (e) in Figure 3. In this study, the SVM classifiers built for regions (c) and (f) performed poorly and were excluded from classifier assembling, which had no impact on the performance of our CAD scheme.

Finally, even the validation method employed in the development of a CAD scheme has an impact on evaluating classification performance. Cross-validation including k-fold cross-validation and leave-one-out (where *k* was set to 1 in a k-fold cross-validation) was a widely used validation technique, which had been proven to have a high variation of accuracy estimates [26]. The most popular validation method with the least bias is the 0.632 bootstrap resampling with replacement, which was used in our work. Both validation methods are applicable for resampling from a small-size database, and the difference between the two methods is whether the sample resampled from the original dataset is replaced back for subsequent resampling. The advantage of the 0.632 bootstrap is notable in that it has the lowest root mean-squared error; when there is only one sample set, the classification performance based on the sample set has a higher chance of being closer to the true performance [27]. Furthermore, with the 0.632 bootstrap, the overfitting may be avoided as effectively as possible due to 100 times (and even more) resampling. In this study, repeating bootstrap resampling 100 times resulted in 100 values of a certain performance index. The standard deviation of the 100 values can be interpreted as the standard error (SE) of the performance index, which is usually smaller than the SE calculated from a single sampling, giving a narrower confidence interval (CI) and a more unbiased estimate of the performance index.

## Conclusion

We have developed a computerized scheme for the detection and differentiation of stage I and stage II pneumoconiosis patients from normal data sets based on the wavelet transform-based energy texture features on DR chest images. The CAD scheme has potential for the early detection of pneumoconiosis.
